# The Role of Islamic Beliefs in Facilitating Acceptance of Cancer Diagnosis

**DOI:** 10.3390/curroncol30090565

**Published:** 2023-08-22

**Authors:** Amina Benidir, Marie-Josée Levert, Karine Bilodeau

**Affiliations:** 1 Faculty of Nursing, University of Montreal, Station Centre-Ville, Montreal, QC H3C 3J7, Canada; mj.levert@umontreal.ca (M.-J.L.); karine.bilodeau.2@umontreal.ca (K.B.); 2 Centre for Interdisciplinary Research in Rehabilitation of Greater Montreal, 6363, Hudson Road, Montreal, QC H3S 1M9, Canada; 3 Maisonneuve-Rosemont Hospital Research Centre, 5415 Boul. Assomption, Montreal, QC H1T 2M4, Canada

**Keywords:** cancer, ethnic minorities, Arab Muslims, couple, religious beliefs

## Abstract

Although survival rates for patients with cancer have increased, this disease continues to affect couples significantly. Religion and culture seem to be part of the therapeutic process for people with cancer. Despite the abundance of the Arab Muslim community in Western countries, there is a lack of documented data on Arab Muslim couples experiencing cancer. A simple exploratory qualitative study was conducted through semi-structured interviews on six married couples (*n* = 12) identifying with the Arab Muslim culture and being affected by cancer. An iterative data analysis was performed. Results were reported under the following themes: accepting illness through coping strategies provided by Muslim religious beliefs and practices, experiencing problems with the expression of needs and feelings within the couple, experiencing closeness within the family, and experiencing illness in the hospital setting as Muslims. Our results show that Islamic beliefs can facilitate acceptance of a cancer diagnosis. It is also noted that religion seems to unite spouses in supporting each other and maintaining hope in a difficult context. Communication issues may persist between a couple due to stressors related to cancer. The results of this study could raise awareness about the importance of exploring religious and spiritual beliefs when supporting couples affected by cancer.

## 1. Introduction

Although survival rates for patients with cancer have increased according to improved curative therapies, the impact of this disease on families persists [1,2,3]. Spouses are the most affected by this disease due to the burden involving their own well-being as well as that of their ill loved one [4,5,6,7]. Among couples with cancer from cultural communities, religious and spiritual beliefs may facilitate acceptance of cancer [8,9,10,11,12]. Religious and spiritual values encourage behaviors that facilitate compliance with cancer treatments [13]. Several authors recommend focusing on the experience of families from an ethnoreligious community [9]. Religious beliefs can be associated with the traditional practices, rituals, or rules derived from a religion. Conversely, spirituals beliefs can be subjective and abstract as they can be defined as a connection with nature or God to find a meaning in life. Moreover, faith can be related to religious and spiritual beliefs and can be associated with the relation with God [14].

For some populations, such as Arab Muslims, families’ and couples’ experiences with cancer are different due to cultural and religious beliefs [15]. The lack of consideration of the cultural needs of this population affects the quality of care [16,17,18]. There is a scarcity of literature available to understand the experience of spouses within Arab Muslim couples affected by cancer. To the best of our knowledge, studies that focus on the experience of Arab Muslim couples with cancer only address the perspective of female patients with breast cancer and disregard the experience of spouses [19,20,21,22,23]. This justifies the need to explore the experience of both spouses within Arab Muslim couples facing cancer, to gather greater information on the subject.

In Canada, the city of Montreal contains the largest Arab Muslim population in the country [24]. In the province of Quebec, immigrant couples often lack support networks, have access to few resources, and deal with difficulties associated with their move to a different country [25]. Given the challenges faced by ethnic minorities in healthcare, it is recommended that healthcare professionals take into consideration the beliefs and needs of patients and their families in order to provide culturally consistent quality care [26,27,28,29,30]. When considering the cultural and religious needs of these individuals, access to fair and equitable care is possible [31,32,33,34,35]. It is recommended that healthcare professionals develop an in-depth knowledge about the influence of culture on their patient’s experience as well as that of their families [36]. Thus, due to the high population of Arab Muslim individuals in Canada, the impact of cancer on spouses, and the lack of studies addressing couples’ experiences with cancer, further research needs to be developed in this sector [2,18,29,37,38,39,40].

This study aims to investigate the impact of religious beliefs, particularly Islamic beliefs, on the process of accepting a cancer diagnosis among couples.

## 2. Materials and Methods

### 2.1. Theoretical Background

A definition and a model were used to explore this study’s phenomenon. First, Purnell’s definition was selected to narrow the meaning of culture. According to Purnell, culture is a set of values, customs, lifestyles, beliefs, and thoughts that shape people’s decisions and worldviews [41]. In addition, the Calgary Family Assessment Model was used to understand the experience of the couples under study [42]. The Calgary Family Assessment Model is based on Systems Theory and proposes that the family is a complex system in which individuals interact with each other on an ongoing basis. Therefore, a change in one family member, such as the diagnosis of a serious illness, affects all family members at different levels [42]. The interview guide for this study was inspired by the Calgary Family Assessment Model.

### 2.2. Study Design

An exploratory qualitative design was employed [43]. This type of design represents the first stage of research in the face of an unknown and under-investigated research problem [43,44]. To recognize the uniqueness of each participant’s experience, the qualitative iterative data analysis proposed by Miles, Huberman, and Saldaña was selected [45]. The project was approved by an institutional research ethics board in Montreal (#2021–2609).

### 2.3. Recruitment and Sampling

The sampling methods used for recruiting participants were accidental sampling [46,47] and snowball sampling [48]. We had several selection criteria: one member of the couple had been diagnosed with cancer and undergoing active treatment (chemotherapy, radiation, surgery, etc.) for the past six months; the couple identified as part of Arab Muslim culture; both members of the couple were able to communicate, write, and read French (at least one member had to be at an intermediate level); and the couple was married. In addition, both members of the couple had to agree to participate in dyadic interviews. The application of the selection criteria brought homogeneity to the recruited participants [49]. The recruitment of the couples in this study was carried out by sharing a recruitment poster via social networks (e.g., Facebook) and in an oncology outpatient clinic located in the city of Montreal.

### 2.4. Data Collection 

Data were collected using a socio-demographic questionnaire and dyadic semi-structured interviews.

#### 2.4.1. Socio-Demographic Questionnaire

Participants completed a socio-demographic questionnaire before the semi-structured interviews began. The questionnaire items provided information on age, level of education, type of employment, marital status, number of children, year of arrival in the province of Quebec (Canada), ethnic origin, branch of Islam of practice, immigration status, and their level of religiosity. Although the classification of religiosity level remains important in the health sciences, it can be nebulous and imprecise [50]. The level of religiosity was measured using a five-point scale. No validated and French-language detailed instrument was identified by the authors; therefore, the five-degree scale utilized was developed by the lead author but inspired by those proposed by numerous authors [51,52,53]. The first degree is “Non-Religious”, and it implies that the individual denies the existence of God and does not practice any fundamentals of Islam (refers to regular reading of the Qur’an, the observance of prayer, fasting, almsgiving, and the observance of the lawful and unlawful acts dictated by the scriptures contained in the Qur’an and in the collection of texts that includes the sayings of the Prophet Muhammad) in their life. The second degree, “Spiritually Awake”, means that the participant fully acknowledges the existence of God and occasionally practices some of the Muslim religion’s fundamentals. The third, “Somewhat Religious”, implies that the participant applies most of the fundamentals of the religion daily. The fourth, “Fairly Religious”, refers to someone who feels that they apply almost all the fundamentals of the religion daily. The final degree, “Highly Religious”, implies that the person’s life is entirely conducted by all the fundamentals of the religion and that they apply them in their life in a rigorous manner.

#### 2.4.2. Dyadic Semi-Structured Interviews

The interviews were conducted from June to October 2021 entirely via videoconference due to COVID-19 pandemic health measures. The mean length was 52 min. The structure of the guide was inspired by the Calgary Family Assessment Model [42]. This model suggests dyadic questions to obtain a deep understanding of the experience of both individuals in the couple [42]. The questions focused on the couple’s experience with cancer, as well as the importance of religious and cultural beliefs about their health experience. Questions were directed to both spouses, and some were designed to understand whether the spouses agreed with each other’s perspectives. A logbook was completed following each interview to allow a thorough description of the phenomena discussed by the participants [45]. This logbook was consulted during the data analysis.

### 2.5. Data Analysis

Following the integral transcription of the interviews, a qualitative analysis of content was conducted, which consisted of an iterative process including data displaying, data presentation, and verification of findings [45]. For data displaying, data were selected, simplified, and transformed. Each participant’s data were first processed individually and then in pairs. For the presentation of the data, the results were displayed in Excel tables and diagrams that present the experience of each couple. For data verification, the data from the different pairs were compared and the authors (AB, KB) made coherent links between themes and sub-themes. For verification, iterations between the preliminary results, data, and codes were performed several times to identify the final themes. To assist the analysis, verbatims were transposed integrally into QDA Miner^TM^ [54]. Data saturation was observed after six interviews. For a qualitative study with a homogenous sample, important themes are often already identified in the first six interviews [49]. Credibility, reliability, and transferability were ensured throughout the research protocol [45]. Concerning the notion of credibility, the authors used rephrasing during the interviews to avoid erroneous conclusions. In this sense, themes, sub-themes, and codes were checked to attest to their authenticity, thus ensuring researcher triangulation [45]. For reliability, the authors ensured consistency between the purpose of this study and the research protocol, including validating the adequacy of the original research question and the process used to answer it. To ensure transferability, the characteristics of the participant sample and recruitment methods were documented [45].

Given the lead author’s identification with Arab Muslim culture, a sensitivity to the research topic could have been raised. To ensure neutrality during the data analysis, the associate authors thoroughly reviewed the codes, themes, and findings and provided feedback regarding the meaning attributed to the participants’ stories by the lead author.

## 3. Results

### 3.1. Participants

Participant characteristics are presented in Table S1. This study’s population was six couples of married individuals (*n* = 12) affected by cancer who identified with the rites, customs, and traditions of Arab culture and the Muslim religion, regardless of the branch of Islam to which they belonged and their level of religiosity. The average age of the participants was 52 years; all the couples had children at the time of the diagnosis; most participants immigrated to Canada more than ten years ago; and two couples were Moroccan, two were Algerian, two were Lebanese, and one was Canadian Moroccan. Within the participants who suffered from cancer, three had breast cancer, one had nasopharyngeal cancer, one had metastatic prostate cancer, and one had Hodgkin’s lymphoma. Most participants were Sunni, and two were Shiites. Regarding the level of religiosity, two participants described themselves as “Spiritually Awake”, three as “Somewhat Religious”, four as “Fairly Religious”, two as “Highly Religious”, and one as between “Fairly Religious” and “Highly Religious”.

### 3.2. Main Themes and Subthemes

**Figure 1:**
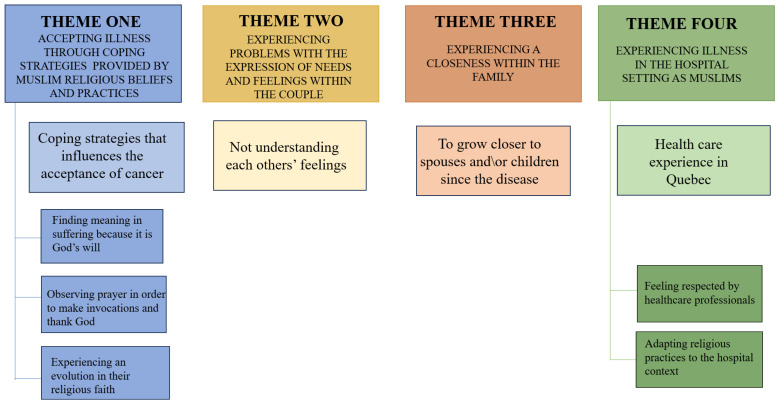
Main themes and sub-themes from interviews.

The results fall within four main themes (Figure 1): (1) accepting illness through coping strategies provided by Muslim religious beliefs and practices; (2) experiencing problems with the expression of needs and feelings within the couple; (3) experiencing a closeness within the family; and (4) experiencing illness in the hospital setting as Muslims.

#### 3.2.1. Theme One: Accepting Illness through Coping Strategies Provided by Muslim Religious Beliefs and Practices

Although the diagnosis of cancer was initially experienced by almost all participants as a shock, their testimonies suggest that certain coping strategies influenced their acceptance of their cancer, or that of their spouse; notably, finding meaning in suffering because it is God’s will, observing prayer in order to make invocations and thank God, and experiencing an evolution in their religious faith.

##### Finding Meaning in Suffering Because It Is God’s Will

A strategy that helped the participants in the physical and psychological suffering caused by cancer is the belief in “God’s destiny” (in Arabic, “Qadr Allah”). This concept encourages believers to accept that the events that make up the present, the past, as well as the future are already determined. Kamal (pseudonyms are used in the text to ensure participants’ confidentiality) (couple 6), who describes himself as “Fairly Religious”, mentioned that a disease could certainly be good, which justifies that God’s intentions should never be questioned:

*[…] Qadr is a notion. For us, it is not to consider an evil, whereas it is a blessing. We don’t know! We must believe in God’s destiny [and accept it]. Whether it is good or bad, according on our perception of good and bad. But the divine perception always has a reason.*

*(Kamal, couple 6)*

Brahim (couple 5), who qualifies himself as “Highly Religious”, specified that he would have been satisfied with the fate that God has destined for him, and would be satisfied with this, whether there was a cure or not. In this sense, this participant reported:

*We must accept God’s fate, so we decided to follow the doctor’s treatment. If there is healing, it is good. If there is no healing, that’s good too. That’s God’s destiny. That’s what we told ourselves.*

*(Brahim, couple 5)*

Cancer was also reported by several participants as a positive event that strengthened their piety. In this context, Sarah (couple 1) said:

*The fact that I accepted the illness and said to myself: it is God, who gave it to me. Yes, I know that it is serious, that it is aggressive, that it can recur. But by doing what is necessary with Him, He is merciful! Today, I am still alive, four years later. I can only thank Him, and thanking Him is not just by saying “Thank you, God”, but by doing things. He told us himself, it’s being in the whole hierarchy of practice.*

*(Sarah, couple 1)*

##### Observing Prayer in Order to Make Invocations and Thank God

Most participants reported performing prayer in order to make requests or thank God. Prayer was reported by Hussain (couple 3), who described himself as “Spiritually Awake”, as the only way to hope and to support his sick wife: “[…] Sometimes you reach a point, like, you can’t do anything else, just pray, just ask God”. Khaoula (couple 4), who qualified herself as “Fairly Religious”, reported praying to thank God for good news, despite her husband’s metastatic cancer:

*So that’s why when I get home, well, I do my prayer. I say to myself: well, I thank God… Nacer had his treatment or his exams, or we had good news from the doctor, for example, after a meeting, so, I do my prayer.*

*(Khaoula, couple 4)*

Zoulikha (couple 5), who revealed that she is “Highly Religious”, explained that she found refuge during prayer moments and allowed herself to become emotional and vulnerable, as well as asking God to heal her husband Brahim:

*During those difficult times, do you know what I used to do? At the time of Dhor (daily prayer marking the start of the afternoon.) or El- Asr (daily prayer marking the end of the afternoon.), I would call upon God in my prayers… All that I had in my heart, I emptied in my prayer… I was praying and crying… I had a feeling that God would not abandon me… I was saying: “Oh God, it is you who created my husband, and it is you who will heal him”. About this, God said: “Ask me, and I will exalt”.*

*(Zulikha, couple 5)*

##### Experiencing an Evolution in Their Religious Faith

Several participants mentioned that the illness had revived their religious faith, and a few had seen their religious practices change throughout this experience. Mohamed (couple 1) reported that from his own perspective, his wife’s faith evolved because of her weakening health. At the same time, Sarah—his wife—said that she became more religious because of the perspective on her life this experience brought her:

*The illness redefines your perspective […] of your own life… let’s say you can die tomorrow. Because when you are healthy, you don’t have the same perspective, you know? Our horizon is not drawn in the same way and we don’t see things in time in the same way. […] It accelerated my way of research and development [religious] that I began slowly.*

*(Sarah, couple 1)*

Some participants stated that their religious practices have become more important in their lives since the cancer. Leila (couple 2) confided, speaking of her husband: “[…] It has increased a lot, let’s say. He has always been a believer, but he wasn’t practicing a lot. So yes, he started to practice religion a lot more since my diagnosis”. Moreover, Khaoula (couple 4) mentioned being more assiduous in her religious practices since Nacer’s illness:

*Since Nacer’s illness, and with all the uncertainty it has brought, I have become more religious. […] Before, I used to do it, to stop it, to do it, to stop it [praying]. But now, it has become like a ritual. I now do my prayer every day, you know. So, yes, the illness has increased my faith a little.*

*(Khaoula, couple 4)*

For Julie (couple 6), who is a convert to Islam and who considers herself “Fairly Religious”, reading and listening to Quranic verses have also been part of her strategies to cope with the disease: “[…] Even though I don’t speak the language and I have difficulty reading Arabic, I have tried to read the Qur’an…to listen to the reading of the first verse, but it’s not easy to understand it…”.

#### 3.2.2. Theme Two: Experiencing Problems with the Expression of Needs and Feelings within the Couple 

Most participants reported experiencing problems with the expression of their needs and their feelings during their disease or that of their spouse. In this sense, some participants testified that they did not feel sufficiently considered by their spouses throughout their cancer. Sarah (couple 1) reported feeling that her husband did not show enough interest in her health condition:

*He never asked me “What are you taking for treatment? Why are you taking this treatment? What does this treatment do?”. He never came up with those questions. So that was a little bit difficult for me because it hurts when I’m at my lowest. I would have liked him to show more interest in my situation.*

*(Sarah, couple 1)*

Leila (couple 2) also reported being affected by what she interpreted as a lack of interest from Hussain:

*He didn’t show his feelings. I thought it was a little bit weird. At one point, it was like, “Okay, he doesn’t care. He doesn’t care!” It’s funny to say, but he never cried. He didn’t really have any reaction. When I announced it [the diagnosis], like, my dad cried, my sisters cried, my brother also. Everyone got emotional. Then, he [my husband] was normal. It was like, “It’ll pass. It’s okay”. Then it had kind of made me angry, like!*

*(Leila, couple 2)*

Half of the spouses interviewed reported hiding their emotions to “protect” or not worry their ill partner. At the same time, these participants also reported a need to share their distress with their spouses. On this point, Hussain stated in response to his wife Leila’s statement:

*I was smiling in front of her and trying to be strong, I supported her and I never talked about how I felt because I didn’t want to worry her. We had to focus on her health, that was the most important thing. [… I would have liked to tell her how I felt, but I didn’t want to hurt her.*

*(Hussain, couple 2)*

#### 3.2.3. Theme Three: Experiencing a Closeness within the Family

Most participants reported that they had grown closer to their spouses since the disease. They reported that their relationship had evolved because of the uncertainty brought on by cancer. This shift was triggered by the recognition of the ordeal they faced. Mahmoud (couple 3) said: “The disease changed my perspective of life, you know. At any time, you can lose somebody without… without knowing”. Leila (couple 2) mentioned: “We became closer because we realize with the disease that everything can change in a second. There is nothing to take for granted in life”. As an answer to his wife (Leila), Hussain mentioned that their relationship has been strengthened:

*Now, our relationship is much deeper. We are much more than two married people. We act as crutches for each other. She can…she can talk to me about anything that’s on her mind, and I can talk to her…Her illness has definitely brought us closer together….*

*(Hussain, couple 2)*

Half of the couples mentioned experiencing closeness with their immediate family members. More specifically, participants reported that their children had grown closer to them since their diagnosis. In this sense, Mahmoud (couple 3) said that their relationship as a couple with their children had grown stronger since Rola’s breast cancer: “And we became closer after her disease. The kids were close to us. Like, we became really bonded family”. Khaoula (couple 4) indicated that she tried to rekindle the bonds with her children who have left the family home:

*The family ties with our two children have become stronger. Even though they are adults, I still tried to strengthen the ties with them through my husband’s illness. We have also benefited from their support, even if they have their own lives.*

*(Khaoula, couple 4)*

#### 3.2.4. Theme Four: Experiencing Illness in the Hospital Setting as Muslims

This theme, which describes participants’ health care experience in Quebec, is subdivided into two sub-themes: feeling respected by health care professionals and adapting religious practices to the hospital context.

##### Feeling Respected by Healthcare Professionals

All participants reported feeling that their religious practices were respected by healthcare professionals during their hospital visits to treat their cancer or their spouse’s cancer. Participants wearing the hijab (refers to the Muslim tradition whereby a woman, after puberty, must cover her head and neck, while leaving her face visible) expressed that their need for modesty was highly considered and respected by healthcare professionals. Sarah (couple 1), who described her level of religiosity as between “Fairly Religious” and “Highly Religious”, said:

*There was always respect. Even in radiotherapy, it was a male radiologist and a male radiation oncologist, and they used to always ask me: “Are you okay?” Because, well, in radiotherapy, you need to get undressed to receive the rays. So they always asked me: “There’s a male resident: are you okay with that? Is that okay?”. I would tell them I was fine with it.*

*(Sarah, couple 1)*

Leila, who described herself as “Somewhat Religious”, revealed appreciating not having to make any request for her modesty to be considered when the nurses installed the needle of a central venous catheter with an implantable chamber:

*In this hospital, when we were receiving our chemo treatments, we were sitting in cubicles separated by a curtain. Because I wear a hijab, I always had to push it back or remove it so they could put the Port-a-Cath needle in. They [the nurses] closed the curtains every time, without me asking for it. Even though there was often no man, they still took the time [to do it]. I really appreciated it, because I realized later that when they put it [the port-a-cath needle] on other people, they didn’t close them [the curtains].*

*(Leila, couple 2)*

##### Adapting Religious Practices to the Hospital Context

Five participants described adjusting their religious practices in hospital settings. This adjustment was reported by participants identifying with various degrees of religiosity. Brahim (couple 5), who identified himself as “Highly Religious”, indicated that he adapted his prayer to the hospital context by shortening his ablutions (religious purification consists of cleansing certain body parts with water. This is a required practice before certain religious acts, notably prayer):

*I always kept my timouma (stone used to perform “dry ablution,” authorized in periods of illness or in cases where access to water is problematic, to facilitate the practice of prayer for Muslims) nearby, so I could pray directly on my [hospital] bed… I didn’t pray in the hallway or anything, so it didn’t attract people’s attention. I wasn’t praying out loud (certain prayers require that the person recite the Qur’an verses out loud, but it is possible to do this quietly, depending on the situation) […] Anyway, I don’t even think people were noticing that I was praying.*

*(Brahim, couple 5)*

Sarah (couple 1) testified removing her hijab and wearing a short-sleeved jacket (according to the Muslim tradition, women wearing the hijab must always cover their “modesty zone” in front of men (other than their husbands, fathers, brothers, and uncles). The “modesty zone” includes the entire body apart from the face, hands, and feet) during hospitalizations: “I don’t wear my hijab when I am hospitalized, and I also agree to wear a jacket”. Khaoula (couple 4), who described herself as “Fairly Religious”, mentioned that she did not pay particular attention to religion during her husband’s hospitalizations, and preferred to carry out her religious practices privately, at home: “I adapt. I adapt myself. So, when I go home, I perform my prayers”.

## 4. Discussion

This study reveals coping strategies provided by Muslim religious beliefs, the effects of cancer on family and couple dynamics, and the experience of hospitalization. To our knowledge, no study had yet explored this phenomenon. In this sense, several interesting findings emerged from the analysis of our results. Our study results propose that religious faith evolves over the course of the couple’s experience. Participants explained that the illness made them aware of the importance of religious practices in their personal lives. Moreover, religion seems to have united the partners during this ordeal, through the mutual hope that was fostered during the illness in addition to the prayers invoking healing. Up to date, no study has clearly described that cancer might lead Muslims to practice their religion more thoroughly. However, findings from a descriptive correlational study conducted in Spain suggest that cancer contributes to the consolidation of beliefs as well as spiritual and religious practices of patients with cancer [55]. This study also presents that religious beliefs can facilitate acceptance of the diagnosis and the prognosis of the disease. Similarly, a qualitative descriptive study conducted on 21 American Christian patients with incurable cancer draws similar results [56]. Participants in this study reported accepting their condition due to several secular beliefs, including those that remind them that death is inevitable, and that God promised a perfect life in Paradise. According to the participants, these facilitating beliefs allowed them to maintain their identity and remain positive, even if they “lost the battle” against the disease. Relatedly, couples in our study reported that, according to their beliefs, the occurrence of the illness is predestined, which facilitated their acceptance. It also appears that religious and spiritual beliefs may help to provide meaning to the cancer experience, develop positive attitudes toward suffering, and reduce the risk of depression [57,58,59]. Different studies conducted in Saudia Arabia, Iran, Turkey, Malaysia, and Tunisia report that the belief in “Qadr Allah” for Muslims facilitates acceptance of a cancer diagnosis by reminding them that their illness was part of God’s plan [60,61,62,63]. A study conducted in Tunisia with women newly diagnosed with breast cancer suggests that the religious coping of the Muslim religion was effective for the participants to accept the diagnosis and thus live with the disease promptly [63]. Prayer was described by participants in our study as an opportunity to formulate demands to God and to experience emotions and moments of vulnerability, and to do so in a non-judgmental setting. From this perspective, studies also represent Muslim prayer as a source of comfort and hope during illness [64]. An Iranian study, which aimed to understand the importance of prayer on patients suffering from cancer, relates that canonical prayers could reduce anxiety, pain, and depressive and physical symptoms of patients with cancer [65].

Our findings also described the dyadic experience of Arab Muslim couples with cancer. Our participants first had trouble expressing their needs within the couple since finding out they had cancer. In this sense, some participants had more difficulty communicating their apprehensions and feelings about their spouse’s illness, leading to a feeling of lack of compassion and support toward the ill spouse. This finding has similarities with the experience of couples outside North America. A Chinese study that explored the experience of couples with colorectal cancer in China and a Norwegian study presenting the experience of couples facing cervical cancer demonstrate similar results [66,67]. In these studies, couples also report insufficient communication that led to avoidance, a perception of little support from the spouse, and limited information sharing. Furthermore, the results of our study conclude that the participants have experienced a closeness within their couple, as well as with their children, since the cancer diagnosis. Thus, the awareness of the precariousness of life encouraged a reinforcement of the spouses’ relationship and family ties. This finding is consistent with those of a study conducted in Malta [68]. In this study, husbands whose wives underwent a mastectomy and radiation treatments to treat their breast cancer testify that their couple’s relationship evolved and that they highly valued the time spent with their wives. Some studies also report that family bonds can become stronger due to the experience of difficult events and the severity of the cancer diagnosis [69,70].

Participants in our study reported feeling respect towards their religious practices from healthcare professionals. This finding is in contradiction with the conclusions of consulted studies in which Muslim patients share discriminatory experiences as well as a lack of consideration given to their religious beliefs in hospital settings [30,71]. Nevertheless, the historical and geopolitical context of the two studies may influence their results. A qualitative study that aimed to explore nurses’ perspectives on the cultural aspects of their Muslim patients, and of the Muslim patients being cared for, was conducted in Spain where the recent immigration of Arab Muslims, especially North Africans, may influence the results and make it difficult to transfer to the Muslim population in other countries [30]. In addition, the Spanish historical context includes events opposing Muslims and Christians, which some experts believe has left persistent Islamophobia in Spain [72]. A descriptive study shows that 22% of Arab Muslims surveyed report discrimination in healthcare facilities in France [71]. Nevertheless, it may be difficult to transfer this study’s conclusions to a Canadian context because of the immigration policies existing in France. Moreover, our conclusions regarding the adjustment of religious practices in the hospital setting coincide with those of a Spanish study [73]. These authors documented flexibility for some Muslim women in receiving care from male health professionals, practicing prayer at home, and removing the hijab during periods of hospitalization, regardless of their degree of religiosity.

Finally, our study results highlight the need for future educational activities or support groups for the minority groups of patients with cancer and their spouses, as well as a continuous education series for multidisciplinary teams of healthcare providers. For instance, creating clear and concise patient booklets/brochures, which will provide culturally appropriate information, could increase the patient’s adherence to medical care, improve response/resilience to stress, and augment the quality of life during each stage of the “cancer journey” for the afflicted patients and their close relatives.

### Strengths and Limitations

One of the strengths of this study is the simultaneous participation of both spouses during the interviews. Consequently, the statements brought by the spouses were directly confirmed or refuted. In this sense, the dyadic interview questions encourage freedom during the discussions within couples in addition to encouraging the sharing of truthful and sincere information between them [74]. This study also provides specific results to the Arab Muslim community, based on the context of this study, which was conducted in the cosmopolitan city of Montreal, the second largest city in Canada. Furthermore, the research design allowed for special consideration of the couple’s narrative, allowing for a more detailed understanding of their experiences. In addition, the lead author’s knowledge of Arab Muslim customs, traditions, and beliefs is a strength of this study. In this sense, Arabic or Quranic lexicon terms were known and could be appropriately incorporated into the results. On another note, there are some limitations that must be considered when interpreting the results. First, several important characteristics of our study sample may limit the transferability of the results to the broader Arab Muslim community. Most of the participants have been established in Montreal for more than 10 years, which makes our findings differ from the reality of Arab Muslim couples with cancer with a precarious immigration status. All the couples interviewed have children, are heterosexual, and are in a narrow age range, which could limit the scope of our findings for gender-diverse or childless Arab Muslim couples who can experience other challenges. Our participants are from Morocco, Algeria, and Lebanon and are predominantly Sunni, which may also limit the transferability of our findings to couples from other Arab countries and practicing other branches of Islam. On another note, it is important to acknowledge the differences in religious practices and beliefs within the same religious or ethnic community. For this reason, our results should be considered sparingly when treating Arab Muslim patients, in order to preserve the uniqueness of each individual from this cultural and religious minority. Due to the COVID-19 pandemic and limited time for the master’s degree, the authors did not conduct post-interview feedback with the participants to validate the themes that emerged from the interviews. 

## 5. Conclusions

This study informs that couples can be challenged by a cancer diagnosis. Furthermore, the participants’ narratives confirm that religious beliefs positively influence hardship in the context of life-threatening illnesses. Healthcare professionals can learn to adapt and understand the influence of religious beliefs, practices, and customs during various life transitions, such as birth, illness, suffering, and death. In addition, religious beliefs appear to be a common thread that spouses use to support each other and maintain hope in a difficult context. In sum, the results of this study can inform about the importance of providing culturally appropriate care to families and couples affected by cancer in order to improve their care experience.

## Data Availability

No new data were created or analyzed in this study. Data sharing is not applicable to this article.

## Supplementary Materials

The following supporting information can be downloaded at: https://www.mdpi.com/article/10.3390/curroncol30090565/s1, Table S1: Participants characteristics, *n* = 12.
