# Supplementary material for: The Role of Islamic Beliefs in Facilitating Acceptance of Cancer Diagnosis
Source: Curr Oncol. 2023 Aug 22;30(9):7789–801. doi: 10.3390/curroncol30090565 (PMC10530149; doi:10.3390/curroncol30090565)
Supplement: Supplementary file 1 [file curroncol-30-00565-s001.zip › curroncol-2501475-supplementary.pdf]

Table S1. Participants' characteristics, *n* = 12.

	Couple 1	Couple 2	Couple 3	Couple 4	Couple 5	Couple 6
**Pseudonyms**	Part. A: Sarah<br>Part B: Mohamed	Part.A: Leila<br>Part.B: Hussain	Part. A: Rola<br>Part. B: Mahmoud	Part. A: Khaoula<br>Part. B: Nacer	Part. A: Zoulikha<br>Part. B: Brahim	Part. A: Julie<br>Part. B: Kamal
**Age**	Part. A: 56<br>Part. B: 60	Part. A: 31<br>Part. B: 30	Part. A: 43<br>Part. B: 50	Part. A: 58<br>Part. B: 66	Part. A: 56<br>Part. B: 60	Part. A: 55<br>Part. B: 55
**Years since immigration**	Part. A: > 10 years<br>Part. B: > 10 years	Part. A: > 10 years<br>Part. B: < 5 years	Part. A: > 10 years<br>Part. B: > 10 years	Part. A: > 10 years<br>Part. B: > 10 years	Part. A: > 10 years<br>Part. B: > 10 years	Part. A: na<br>Part. B: > 10 years
**Years of immigration**	28	3	20	34	28	24
**Number of children**	3	1	3	2	4	2
**Country of birth**	Part. A: Algeria<br>Part. B: Algeria	Part.A: Lebanon<br>Part.B: Lebanon	Part.A: Lebanon<br>Part.B: Lebanon	Part. A: Algeria<br>Part. B: Algeria	Part. A: Morocco<br>Part. B: Morocco	Part. A: Canada<br>Part. B: Morocco
**Branch of Islam of practice**	Part. A: Sunnism<br>Part.B: Sunnism	Part. A: Sunnism<br>Part.B: Sunnism	Part. A: Sunnism<br>Part.B: Sunnism	Part. A: Sunnism<br>Part.B: Sunnism	Part. A: Sunnism<br>Part.B: Sunnism	Part. A: Sunnism<br>Part.B: Sunnism
**Level of religiosity**	Part. A: between fairly religious and highly religious<br>Part B: Fairly religious	Part. A: Fairly religious<br>Part. B: spiritually awake	Part. A: Somewhat religious<br>Part. B: Somewhat religious	Part. A: Fairly religious<br>Part. B: spiritually awake	Part. A: Highly religious<br>Part. B: Highly religious	Part. A: Fairly religious<br>Part. B: Highly religious
**Immigration status**	Part. A: Canadian citizen<br>Part. B: Canadian citizen	Part. A: Canadian citizen<br>Part. B: Canadian citizen	Part. A: Canadian citizen<br>Part. B: Canadian citizen	Part. A: Canadian citizen<br>Part. B: Canadian citizen	Part. A: Canadian citizen<br>Part. B: Canadian citizen	Part. A: Canadian citizen<br>Part. B: Canadian citizen
**Participant diagnosed with cancer**	Part. A: Sarah	Part. A: Leila	Part. A: Rola	Part. B: Nacer	Part. B: Brahim	Part. A: Julie
**Type of cancer**	Breast cancer	Hodgkin's lymphoma	Breast cancer	Prostate cancer with bone metastasis	Nasopharyngeal	Breast cancer
**Stage of cancer at diagnosis**	I	IV	III	IV	IV	I
**Treatments received**	Chemotherapy<br>Radiotherapy<br>Surgery	Chemotherapy	Surgery<br>Hormonotherapy	Radiotherapy<br>Surgery<br>Hormonotherapy	Chemotherapy<br>Radiotherapy	Chemotherapy<br>Hormonotherapy<br>Targeted therapy<br>Surgery
